# The Association between Invasive Group A Streptococcal Diseases and Viral Respiratory Tract Infections

**DOI:** 10.3389/fmicb.2016.00342

**Published:** 2016-03-21

**Authors:** Andrea L. Herrera, Victor C. Huber, Michael S. Chaussee

**Affiliations:** Division of Basic Biomedical Sciences, The Sanford School of Medicine of the University of South DakotaVermillion, SD, USA

**Keywords:** *Streptococcus pyogenes*, group A streptococcus, influenza

## Abstract

Viral infections of the upper respiratory tract are associated with a variety of invasive diseases caused by *Streptococcus pyogenes*, the group A streptococcus, including pneumonia, necrotizing fasciitis, toxic shock syndrome, and bacteremia. While these polymicrobial infections, or superinfections, are complex, progress has been made in understanding the molecular basis of disease. Areas of investigation have included the characterization of virus-induced changes in innate immunity, differences in bacterial adherence and internalization following viral infection, and the efficacy of vaccines in mitigating the morbidity and mortality of superinfections. Here, we briefly summarize viral-*S. pyogenes* superinfections with an emphasis on those affiliated with influenza viruses.

## Introduction

The study of microbial pathogenesis typically examines a single pathogen in an experimental system. The reductionist approach has proven to be highly informative and forms the foundation of our understanding of pathogenesis. Nonetheless, humans are often exposed to an assortment of viral and bacterial pathogens and the factors determining the outcome of these host-pathogen interactions are less well characterized. Viral infections complicated by a concurrent, or subsequent, bacterial infection are known as coinfections or superinfections, respectively. Due to synergistic effects, the mortality of these infections is often greater than that of either the virus or the bacteria alone (Morens and Taubenberger, 2015).

*Streptococcus pyogenes*, the group A streptococcus (GAS), can colonize humans asymptomatically or cause infections of the skin and pharynx. In severe cases, the gram-positive pathogen can cause a variety of life threatening invasive diseases (iGAS diseases) including bacteremia, toxic shock syndrome, pneumonia, and necrotizing fasciitis (the so-called “flesh eating disease”). GAS causes approximately 700 million infections annually, including 1.8 million invasive infections. The mortality rate of these infections is ~20% (Carapetis et al., 2005), despite the availability of antibiotics that are effective *ex vivo*. Here we briefly summarize the epidemiological reports supporting the idea that upper respiratory tract viral infections contribute to the development of iGAS diseases and review studies aimed at identifying the molecular underpinnings of viral-GAS pathogenesis.

## The contribution of varicella, morbilla virus, and Epstein–Barr virus to IGAS diseases

The association between chickenpox and GAS necrotizing fasciitis was described as early as the nineteenth century and varicella continues to be an important contributor to iGAS diseases among children, which is most evident in regions of the world that choose not to vaccinate against chickenpox or that lack the resources to do so (Wilson et al., 1995; Schreck et al., 1996). GAS is the most common bacterial cause of varicella coinfections (Aebi et al., 1996), which typically results in necrotizing fasciitis, bacteremia, and toxic shock syndrome. Usually these diseases occur as the result of infected skin lesions, which provide portals for bacterial entry into the blood. However, varicella-GAS superinfections can also occur, albeit rarely, when varicella skin lesions are not apparent suggesting that the viral infection causes systemic decreases in innate immunity (Laskey et al., 2000; Sivakumar and Latifi, 2008). Prior to the use of the vaccine, the incidence of varicella-GAS coinfections was estimated to be between 15 and 36% (Laupland et al., 2000; Zachariadou et al., 2014). Results of a study that spanned both the pre and post-varicella vaccination periods (1993–2001) showed that the incidence of these coinfections declined from 27% prior to 1995 to just 2% after the availability of the vaccine (Patel et al., 2004).

Both GAS and Epstein–Barr virus (EBV) are common causes of pharyngitis. Two studies focused on patients 25 years-of-age or younger and determined the incidence of EBV-GAS coinfection to be 18 and 29% (Henke et al., 1973; Rush and Simon, 2003). In contrast, < 3% of college students with EBV were also infected with GAS (Chretien and Esswein, 1976). While there are sporadic and infrequent reports of children with infectious mononucleosis developing iGAS diseases, including streptococcal toxic shock syndrome (Pontes and Antunes, 2004) and GAS bacteremia (DuBois and Baehner, 1979), a causal role for EBV in iGAS diseases remains unclear (Watanabe et al., 2012).

Measles virus (MV) infection can also enhance host susceptibility to bacterial infections (de Vries et al., 2012). While the incidence of MV-GAS coinfections is currently low, this has not always been the case. In 1917, an epidemic of severe GAS diseases coincided with a measles epidemic among U.S. Army personnel resulting in bronchopneumonia, empyema, and bacteremia, which were associated with significant mortality (Morens and Taubenberger, 2015). While GAS is currently a relatively uncommon cause of MV superinfections, sporadic cases still occur (Gremillion and Crawford, 1981; Duke and Mgone, 2003; Gahr et al., 2014).

## The contribution of influenza to IGAS diseases

The most thoroughly characterized bacterial superinfections are those involving the influenza virus (McCullers and Rehg, 2002; Peltola and McCullers, 2004). *Streptococcus pneumoniae, Staphylococcus aureus, Haemophilus influenzae*, and GAS are causes of “excess mortality” due to influenza coinfections and superinfections (Peltola and McCullers, 2004; Brundage, 2006), which were first described nearly a century ago (Laennec, 1923). An analysis of lung biopsies from the 1918 influenza pandemic revealed that *S. pneumoniae* and GAS were the most frequently observed bacteria and together contributed substantially to the deaths of approximately 50 million people (Morens and Fauci, 2007). The surprisingly high incidence of cases involving GAS was likely due to the circulation of particularly virulent GAS strains and high carriage rate during this time (Morens and Taubenberger, 2015). More recently during the 2009 H1N1 influenza pandemic, 27% of fatalities among people with laboratory confirmed IAV-bacterial coinfections were caused by GAS^1^. A report of 10 patients in California with laboratory confirmed H1N1-GAS infections during this pandemic revealed that despite treating at least 6 patients with both antivirals and antibiotics, 7 of the 10 patients died and the median age of the deceased was 37 (Jean et al., 2010).

In addition to these cases in the US, iGAS diseases associated with IAV occur Worldwide. In England, the incidence of all iGAS diseases increased by 26% between July 2009 and January 2011 with the highest increase reported in December 2010, which was attributed to an increase in influenza during the same time period (Zakikhany et al., 2011). A retrospective study conducted in France found that one third of children infected with GAS also had laboratory confirmed influenza infection (Parola et al., 2011). In Israel during the 2009–2010 influenza pandemic there was an increase in GAS bacteremia (Tasher et al., 2011). Similarly, the incidence of iGAS diseases in Sweden increased markedly in 2012 compared to previous years, which was attributed to an increase in respiratory viral infections, particularly influenza (Darenberg et al., 2013).

A study of military recruits in the U.S. provides a different perspective illustrating the contribution of IAV to GAS diseases. The recruits were less likely to experience any GAS disease if they were vaccinated against influenza compared to unvaccinated controls, and protection against GAS diseases ranged from 50 to 77% depending on the year of the study (Lee et al., 2008).

In addition to IAV, influenza B virus (IBV) is associated with iGAS diseases (Aebi et al., 2010). In a study conducted in Canada, 8% of patients with iGAS diseases occurring between 1996 and 2008 were also infected with IBV (Allard et al., 2012). In a study conducted in England, 14 of 19 cases of iGAS diseases including bacteremia, septic arthritis, endometritis, and pneumonia had evidence of a previous influenza-like infection and 26% had laboratory confirmed influenza infections, mostly IBV. Significantly, ten of the patients died even though 9 of those 10 were being treated with antibiotics that are effective against GAS *ex vivo* (Scaber et al., 2011).

Collectively, these clinical observations indicate that a substantial number of iGAS diseases are associated with influenza. Moreover, despite the availability of influenza vaccines, the practice of prophylactically treating severely ill influenza patients with antibiotics (Mandell et al., 2007), and the fact that all isolates of GAS are sensitive to penicillin *ex vivo*, the mortality rate of influenza-GAS superinfections is substantial.

## Mechanisms of IAV-GAS superinfection

The effect of influenza virus infection on host susceptibility to bacterial infections has been most thoroughly studied with *S. pneumoniae* and several excellent reviews on this topic are available (McCullers, 2006, 2014; Metzger and Sun, 2013; Rynda-Apple et al., 2015). Influenza infections increase host susceptibility to bacterial infections in a variety of ways including an increase in bacterial adherence after the removal of sialic acid from respiratory cells by viral neuraminidase (McCullers and Bartmess, 2003; McCullers, 2014); destruction of the mucocilliary escalator by viral induced lysis of respiratory epithelial cells (Ohashi et al., 1991; Pittet et al., 2010); decreased expression by alveolar macrophages of the scavenger receptor MARCO following influenza induction of IFN-γ (Sun and Metzger, 2008); depletion of resident alveolar macrophages following influenza infection (Ghoneim et al., 2013). As a result, macrophage-mediated clearance of bacteria is diminished.

Comparatively little is known about the host-pathogen interactions of IAV-GAS superinfection; however, the areas that have been investigated include the development of murine models of superinfection, characterization of viral-induced changes in bacterial adherence and internalization, and assessments of the efficacy of vaccines targeting either the virus or bacteria in decreasing the morbidity and mortality associated with superinfections.

### Murine models of IAV-GAS superinfection

Murine models of IAV-GAS superinfection typically use sub-lethal intranasal inoculations of IAV and GAS, which are separated by 2–7 days. These studies are summarized in Table 1. As is likely the case with human disease, the outcome of murine IAV-GAS infections varies greatly depending on the specific viral and bacterial strains used in the study. In one case, the outcome of an IAV-GAS coinfection was compared between mice inoculated with isolates of GAS from patients with toxic shock syndrome and those from non-invasive infections using an interval of 2 days between viral and bacterial inoculations (Okamoto et al., 2003). Coinfection with isolates from cases of toxic shock syndrome resulted in the death of 80–95% of the mice while inoculation with the same dose of the isolates in the absence of antecedent viral infection resulted in no mortality. In contrast, when mice were coinfected with GAS isolates from non-invasive infections the mortality was < 30% (Okamoto et al., 2003). A subsequent study used a 5 day interval between IAV and GAS inoculations, which allows mice time to begin to recover from the viral infection prior to being infected with bacteria. Using this model, the contribution of eight different isolates of IAV to the outcome of superinfection was determined with a single isolate of GAS (Weeks-Gorospe et al., 2012). The mortality varied between 100 and 20% indicating that the outcome of IAV-GAS superinfections is also affected by the particular type of influenza virus (Weeks-Gorospe et al., 2012). Together, the results indicate that the severity of IAV-GAS superinfections is likely to vary considerably depending on the specific types of IAV and GAS circulating in the population.

**Table 1:** **Murine models of IAV-GAS superinfection**.

**Influenza isolate**	**GAS isolate**		**Superinfection Outcome (% mortality)**	**Pathogenic differences**	**References**
**Superinfection studies:**					
IAV A/FM/1/47 (H1N1)	STSS isolates Non-invasive isolates		80–95% < 30%	IAV increases GAS adherence and internalization in A549 and MDCK cells	Okamoto et al., 2003
8 different Influenza isolates	MGAS315 (serotype M3)		20–100%	Different isolates of IAV variably influence superinfection outcomes	Weeks-Gorospe et al., 2012
IAV A/FM/1/47 (H1N1)	SSI-9 (serotype M1) SSI-9 mutants		90% 20–60%	GAS virulence factors contribute to superinfection outcomes	Okamoto et al., 2004b
**Vaccination studies:**		**Vaccine description**			
IAV A/FM/1/47 (H1N1)	SSI-1 (serotype M3)	Formalin-inactivated IAV	22% (vaccinated) 85% (unvaccinated)		Okamoto et al., 2004a
A/HK/1/68 (H3N2)	MGAS315 (serotype M3)	IIV^a^ and LAIV^b^	22% (LAIV) 33% (IIV) 84% (unvaccinated)		Chaussee et al., 2011
A/HK/1/68 (H3N2)	MGAS315 (serotype M3)	GAS M protein^c^	0% (vaccinated) 42% (unvaccinated)		Klonoski et al., 2014

a *Inactivated influenza vaccine*.

b *Live attenuated influenza vaccine*.

c *Recombinant hexavalent S. pyogenes M protein vaccine*.

The mortality of IAV-GAS superinfections in mice is due, in part, to increased persistence of the bacteria in the lungs of superinfected mice compared to mice infected with only GAS (Chaussee et al., 2011). Persistence is associated with subsequent bacteremia and multi-organ infection. As a result of dissemination, ~10% of superinfected mice develop necrotizing fasciitis (Okamoto et al., 2003).

Since the respiratory tract is altered during IAV infection, including changes in cell types, cytokine milieu, host protein composition and abundance, and endothelial and effector cell functions, it is of interest to know if GAS virulence factors important in a GAS monoinfection are similarly important in the context of a coinfection or superinfection. To address this issue, a serotype M1 strain of GAS and several mutant derivatives were analyzed with a murine H1N1 superinfection model (Okamoto et al., 2004b). Strains included those with mutations in the *hasA* gene (encoding the first enzyme involved in the synthesis of the hyaluronic acid capsule), *slo* (encoding the secreted thiol-activated cytolysin SLO), *speB* (encoding a secreted cysteine protease), *sagA* (encoding a secreted cytolysin SLS), and *mga* (encoding a global transcriptional regulatory protein; Okamoto et al., 2004b). Only 10% of mice coinfected with the parental GAS strain survived. In contrast, between 40 and 80% of mice coinfected with the mutant derivatives survived. The *hasA* gene had the most significant contribution to disease, which was attributed to decreased bacterial adherence due to the loss of the capsule (Okamoto et al., 2004b), which is an adhesion (Schrager et al., 1998). However, the capsule is also antiphagocytic, which likely contributed to the ability of the parental strain, but not the *hasA* mutant, to persist in the lungs of coinfected mice. In this respect, the *hasA* mutant had the fewest bacteria in the lungs of superinfected mice 24 h after inoculation compared to the wild-type strain and the other mutants. The coinfection studies were completed using an interval of 2 days between viral and bacterial inoculations (Okamoto et al., 2004b). The results indicate that virulence factors important in a monoinfection also contribute to coinfection and it will be of interest to determine if similar results are obtained using a superinfection model with a longer interval between viral and bacterial inoculations.

### Adherence and internalization

IAV infection increases the abundance of fibrinogen (Fg) in the respiratory tract beginning approximately 4 days after infection (Keller et al., 2006). The binding of GAS to Fg has been recognized for decades as being important for GAS adherence and internalization within host epithelial cells and for conferring resistance to phagocytosis (Cunningham, 2000). IAV infection enhanced the adherence of GAS to cultured Madin–Darby canine kidney (MDCK) cells in an Fg-dependent manner (Sanford et al., 1982). The finding was confirmed by a subsequent study showing that both bacterial adherence and internalization were enhanced in cells infected with IAV, and that the effect was also dependent on the GAS M protein, which binds to a variety of host proteins including Fg (Hafez et al., 2010). In addition to Fg, *Streptococcus dysgalactiae* binds directly to IAV hemagglutinin (HA) localized to the surface of infected cells. Binding was diminished by the addition of GAS, which indicated that GAS was also likely to bind directly to viral HA (Pan et al., 1979). In this regard, IAV infection of cultured cells (A549 and MDCK) increased GAS adherence and internalization compared to uninfected cells (Okamoto et al., 2003) and GAS bound directly to progeny virus budding from infected cells, presumably by binding to viral HA (Okamoto et al., 2003). Moreover, treating superinfected mice intravenously with antibodies against HA decreased the number of streptococci in the lungs as well as other organs and mortality (Okamoto et al., 2003). Similarly the adherence and internalization of a GAS serotype M6 isolate (JRS4) to MDCK cells was enhanced 4- and 20-fold, respectively, in viral infected cells compared to uninfected cells (Hafez et al., 2010). Both adherence and internalization were dependent on the M6 protein, and both were enhanced by host Fg and fibronectin (Fn) (Hafez et al., 2010).

The influenza neuraminidase cleaves sialic acid, which exposes host proteins involved in pneumococcal adherence (McCullers and Bartmess, 2003). Neuraminidase also stimulates the expression of bacterial receptors by activating TGF-β (Li et al., 2015), which also increases the production of Fn (Schultz-Cherry and Hinshaw, 1996). Similar to Fg, Fn is an important ligand for GAS adherence (Beachey and Courtney, 1989) and internalization (Chaussee et al., 2000; Wang et al., 2006). Using TGF-β knockout mice, Li et al. found that GAS colonization of the lungs 3 days after IAV infection was less than that observed with wild-type mice (Li et al., 2015). Thus several studies using a variety of different isolates of virus and bacteria indicate that IAV infection increases both GAS adherence and internalization by binding to viral HA on the infected cell surface and following increases in the abundance of, and access to, bacterial receptors and the GAS ligands Fg and Fn.

### Vaccination

Vaccination of mice against IAV provides significant protection against mortality from GAS secondary infections (Okamoto et al., 2004a; Chaussee et al., 2011). In one study, mice were vaccinated against influenza and subsequently infected with an H1N1 virus 2 days prior to infection with GAS; only 15% of unvaccinated mice survived compared to 78% of vaccinated mice (Okamoto et al., 2004a). A subsequent study compared the efficacy of live attenuated and killed influenza vaccines in a superinfection model in which mice were inoculated intranasally with a normally sub-lethal dose of GAS 7 days after IAV infection (Chaussee et al., 2011). Morbidity (measured by weight loss) following IAV infection was undetectable among mice vaccinated with either formulation, consistent with protection against the virus (Chaussee et al., 2011). Nonetheless, 22 and 33% of mice receiving the attenuated or killed vaccine, respectively died as a result of GAS superinfection. Thus, the influenza vaccines prevented viral illness and decreased the mortality associated with IAV-GAS superinfections; however, they did not provide sterilizing immunity nor did they abrogate mortality. The results suggest that IAV infection or exposure, even among vaccinated people, may still contribute to the incidence of iGAS diseases.

A related study examined the effectiveness of vaccinating against GAS using a hexavalent polypeptide corresponding to surface localized M proteins (Dale, 1999). In contrast to vaccination against influenza and despite signs of illness after IAV infection, vaccinated mice were completely protected from mortality (Klonoski et al., 2014). One possible explanation for the difference in protection between influenza vaccines and the GAS vaccine is that vaccination against GAS may confer the host with the ability to clear the bacteria by an antibody-dependent mechanism that is not compromised by antecedent IAV infection. In contrast, following vaccination against IAV viral exposure may still impair host defenses sufficiently to promote bacterial superinfection, even in the absence of a clinically apparent viral infection. Interestingly, a recent report using a conjugate vaccine against *S. pneumoniae* showed that this vaccine did not result in 100% protection as obtained with the anti-M protein vaccine (Metzger et al., 2015). This indicates that there is still much to learn regarding the differences in the pathogenesis of IAV-bacterial superinfections and the contributions of vaccine-induced immunity in the context of polymicrobial infections. Future studies focused on defining the mechanisms of vaccine-induced protection will aid in the interpretation of these observations and guide the design of new therapeutic strategies to mitigate the morbidity and mortality of IAV superinfections.

## Summary

GAS causes several life threatening invasive diseases including pneumonia, bacteremia, necrotizing fasciitis, and toxic shock syndrome. Decades of study with GAS in monoculture and with monoinfected animal models has yielded significant insights into the virulence factors and host responses that contribute to invasive diseases (Cunningham, 2000; Fieber and Kovarik, 2014; Walker et al., 2014). Similarly, genomic studies conducted during the past decade have led to insights into the genetic characteristics of GAS isolates from invasive infections (Musser and Shelburne, 2009). A less well understood contributor to iGAS diseases is antecedent, or concurrent, viral infection of the upper respiratory tract. Epidemiological studies and clinical reports support the idea that several viruses, and particularly influenza, increase the incidence of iGAS diseases. Although comparatively little is known about the molecular bases underlying the observations, virus-induced changes in bacterial adherence and effector cell-mediated clearance appear to be particularly important. Future studies will likely identify additional host and bacterial factors important in the development of viral associated iGAS diseases and polymicrobial diseases in general. Such knowledge is especially important in the design of new strategies to control the mortality of influenza-GAS superinfections, which is significant despite the availability of influenza vaccines, anti-viral treatments, and the *ex vivo* susceptibility of GAS to penicillin.

## Author contributions

All authors listed, have made substantial, direct and intellectual contribution to the work, and approved it for publication.

### Conflict of interest statement

The authors declare that the research was conducted in the absence of any commercial or financial relationships that could be construed as a potential conflict of interest.

## Footnotes

^1^Bacterial coinfections in lung tissue specimens from fatal cases of 2009 pandemic influenza A (H1N1) - United States, May-August 2009. Morb. Mortal. Wkly. Rep. 58, 1071–1074.
